# The role of neutrophilia in hyperlactatemia, blood acidosis, impaired oxygen transport, and mortality outcome in critically ill COVID-19 patients

**DOI:** 10.3389/fmolb.2024.1510592

**Published:** 2025-01-06

**Authors:** Basma A. Yasseen, Aya A. Elkhodiry, Hajar El-sayed, Mona Zidan, Azza G. Kamel, Mohamed A. Badawy, Marwa S. Hamza, Riem M. El-Messiery, Mohamed El Ansary, Engy A. Abdel-Rahman, Sameh S. Ali

**Affiliations:** ^1^ Research Department, Children’s Cancer Hospital Egypt, Cairo, Egypt; ^2^ Department of Clinical Pharmacy Practice, Faculty of Pharmacy, The British University in Egypt, Cairo, Egypt; ^3^ Infectious Disease Unit, Internal Medicine Department, Faculty of Medicine, Cairo University, Cairo, Egypt; ^4^ Department of Intensive Care, Faculty of Medicine, Cairo University, Cairo, Egypt; ^5^ Pharmacology Department, Faculty of Medicine, Assuit University, Assuit, Egypt

**Keywords:** lung damage, COVID-19 mortality, neutrophils, lactate, acidosis

## Abstract

**Introduction:**

COVID-19 severity and high in-hospital mortality are often associated with severe hypoxemia, hyperlactatemia, and acidosis, yet the key players driving this association remain unclear. It is generally assumed that organ damage causes toxic acidosis, but since neutrophil numbers in severe COVID-19 can exceed 80% of the total circulating leukocytes, we asked if metabolic acidosis mediated by the glycolytic neutrophils is associated with lung damage and impaired oxygen delivery in critically ill patients.

**Methods:**

Based on prospective mortality outcome, critically ill COVID-19 patients were divided into ICU- survivors and ICU-non-survivors. Samples were analyzed to explore if correlations exist between neutrophil counts, lung damage, glycolysis, blood lactate, blood pH, hemoglobin oxygen saturation, and mortality outcome. We also interrogated isolated neutrophils, platelets, and PBMCs for glycolytic activities.

**Results:**

Arterial blood gas analyses showed remarkable hypoxemia in non-survivors with no consistent differences in PCO_2_ or [HCO_3_^−^]. The hemoglobin oxygen dissociation curve revealed a right-shift, consistent with lower blood-pH and elevated blood lactate in non-survivors. Metabolic analysis of different blood cells revealed increased glycolytic activity only when considering the total number of neutrophils.

**Conclusion:**

This indicates the role of neutrophilia in hyperlactatemia and lung damage, subsequently contributing to mortality outcomes in severe SARS-CoV-2 infection.

## 1 Introduction

Since the emergence of COVID-19 in late 2019, the virus has led to over 776 million confirmed cases and more than 7 million deaths worldwide, leaving a profound and lasting impact (COVID-19 deaths, 2024). While many cases are mild or asymptomatic, a significant proportion of patients develop severe illnesses characterized by acute respiratory failure, multiple organ dysfunction, and systemic inflammation (Berlin et al., 2020; Tan et al., 2021). Given the high mortality rate among these severe cases, understanding the underlying mechanisms that lead to often fatal outcomes in patients remains a critical focus for ongoing research (Grasselli et al., 2021).

Hypoxemia, frequently observed in COVID-19 patients, indicates significant disturbances in both lung and systemic respiratory and circulatory functions, contributing to higher mortality rates. It is typically assumed that hypoxemia results from either defects in oxygen delivery or impaired oxygen-carrying capacity (Xie et al., 2020). A reduction in blood pH, or acidosis, leads to a rightward shift in the oxygen-hemoglobin dissociation curve, causing hemoglobin to release oxygen more readily to tissues. Although this mechanism can be beneficial, it may also result in lower oxygen saturation as hemoglobin retains less oxygen. In the context of COVID-19, where metabolic acidosis may arise due to inflammation and respiratory distress, the increased demand for oxygen compounds these challenges. As blood pH decreases, this imbalance can exacerbate tissue hypoxia and hinder oxygen delivery to vital organs (Kim et al., 2023). Hypoxia and acidosis reinforce each other in a vicious cycle; a drop in blood plasma pH by less than 0.2 units significantly reduces hemoglobin’s ability to bind oxygen. Tissue hypoxia triggers a switch to anaerobic glycolysis, leading to increased lactate production; hyperlactatemia is often regarded as an indicator of hypoxia in critically ill patients (Nechipurenko et al., 2021). Conversely, lactic acidosis—characterized by elevated lactic acid levels in the bloodstream—is associated with increased morbidity and mortality (Carpenè et al., 2022; Gupta, 2022). Evidence shows that blood lactate levels are higher in patients suffering from severe COVID-19 than in those with milder cases (Chen et al., 2020; Kalabin et al., 2021; Velavan et al., 2021), and among hospitalized patients, lactate levels were found to be the highest in non-survivors [reviewed in: (Carpenè et al., 2022):]. However, Iepsen et al. argue that elevated lactate may not directly correlate with tissue hypoxia but instead reflect mitochondrial dysfunction and high adrenergic stimulation, (Iepsen et al., 2022), implying that the causes of increased lactate levels in severe COVID-19 may be complex and not exclusively related to tissue oxygen deprivation.

The liver and kidney dysfunction associated with COVID-19 may impair the ability of these organs to eliminate lactate from thebloodstream, resulting in amplified lactate levels in severe COVID-19 patients (Pagano et al., 2023). Furthermore, neutrophilia—an increase in the number of neutrophils in the blood—occurs in up to 70% of COVID-19 patients and correlates with more severe symptoms (Loyer et al., 2022). Neutrophils are rapidly recruited to the lungs, accumulating in significant numbers within the lung interstitium and alveolar spaces (Castanheira and Kubes, 2023). It has been suggested that activated immune cells, including neutrophils and macrophages in the lungs, may be a major source of lactate released during septic shock [reviewed in (Certo et al., 2021)], particularly when sepsis is complicated by acute respiratory distress syndrome (ARDS) (Matthay and Zemans, 2011). The current study aims to explore whether neutrophilia itself contributes directly to elevated circulating lactate levels in critically ill COVID-19 patients, especially non-survivors.

Mitochondrial impairment has been associated with COVID-19, leading to dysfunctional oxygen utilization and inefficient glucose metabolism through oxidative phosphorylation. This impairment drives a metabolic shift toward anaerobic glycolysis, which increases lactate production. Consequently, elevated blood lactate levels are often observed in patients with severe COVID-19, reflecting the metabolic stress experienced by affected cells (Iepsen et al., 2022). Studies investigating metabolic alterations in severe COVID-19 patients report conflicting results regarding neutrophil metabolism. For instance, some studies have found that neutrophils from severe patients exhibit enhanced glycolysis and defective mitochondrial function (Borella et al., 2022). Moreover, McElvaney et al. have shown elevated cytosolic levels of an enzyme (pyruvate kinase M2), which catalyzes the final step of glycolysis, alongside increased lactate in neutrophils isolated from severe COVID-19 patients, suggesting enhanced glycolytic activity (McElvaney et al., 2020). Conversely, other studies have indicated diminished glycolytic flux and a shift to oxidative metabolism in neutrophils from COVID-19 patients with ARDS (Reyes et al., 2021). This study aims to investigate the metabolic profile of neutrophils, lung damage, and blood acidosis concerning mortality outcomes in a cohort of severe COVID-19 patients.

## 2 Materials and methods

### 2.1 Study design and participants

This is a prospective observational cohort study conducted to assess patients who tested positive for COVID-19 through an RT-PCR test. Patient recruitment took place at the ICU facility within Kasr Alainy Cairo University Hospital - Internal Medicine Quarantine Hospital. Standard supportive therapy, including supplemental oxygen and symptomatic management, was administered as clinically indicated. Patients exhibiting moderate to severe hypoxia, defined as requiring a fraction of inspired oxygen (FiO_2_) of ≥40%, were transferred to the intensive care unit (ICU) for escalated care, including invasive mechanical ventilation when deemed necessary. Within the ICU patient cohort, individuals were categorized into two groups based on their subsequent mortality outcomes: those who survived (ICU-S) and those who died within 20 days of sample collection (ICU-NS). Samples were collected during the period of October 2020 to December 2021 from ICU patients. Individuals with mild to moderate clinical symptoms of COVID-19 or with respiratory symptoms not related to SARS-CoV-2 (negative for SARS-CoV-2) were excluded from the study. All available samples and analyses were conducted by blinded operators and included in the final dataset.

### 2.2 Radiological assessment of lung damage

A lung CT damage score for each patient was calculated according to a semi-quantitative CT severity scoring protocol proposed by Pan et al. (2020). This scoring system is based on the extent of parenchymal involvement in each of the five lobes (Elmokadem et al., 2022) as follows (0) no involvement; (1) <5% involvement; (2) 5%–25% involvement; (3) 26%–50% involvement; (4) 51%–75% involvement; or (5) >75% involvement. The resultant total CT score is the sum of the obtained lobar scores and ranges from 0 to 25.

### 2.3 Blood sample collections, handling, and processing for blood cells isolation and analyses

A volume of 10 mL of fresh blood was collected from all subjects in Acid Citrate Dextrose (ACD) tubes (Greiner Bio-One GmbH, Kremsmünster, Austria). For flow cytometry measurements, 2 mL of collected citrated whole blood was incubated for 15 min with RBCs lysis buffer composed of NH_4_Cl (ammonium chloride), NaHCO_3_ (sodium bicarbonate), and EDTA (disodium) (Don-Doncow et al., 2019). The remaining 8 mL of blood was used for blood cell isolation as previously described (Abdel-Rahman et al., 2021). Briefly, whole blood was centrifuged at 300 × g for 15 min at 25°C with no breaks. (1) The upper clear layer (platelet-rich plasma) was transferred to a new tube and centrifuged to get platelets pellet and platelets poor plasma as described previously (Yasseen et al., 2022). (2) The lower layer was gently layered over equal volumes of lymphocyte separation medium (1.119 and 1.077). Tubes were then centrifuged at 500 × g for 35 min at 25°C with no brakes and lowered acceleration. After centrifugation, the second layer containing peripheral blood mononuclear cells (PBMCs) was collected, washed in PBS, and the pellet was suspended in 100 µL PBS. The layer comprising neutrophils was collected and washed in Hank’s Balanced Salt Solution (HBSS), then centrifuged again at 350 × g for 10 min at 25°C. The supernatant was discarded, and the pellet was suspended in RBCs lysis buffer and incubated at room temperature for 15 min. Following centrifugation for 10 min at 350 × g, the pellet was suspended in 100 µL PBS. Neutrophils and PBMCs counts were performed manually by a hemocytometer. Cellular viability was monitored for each sample using trypan blue, and only cells that exhibited more than 90% viability were used in the analysis.

### 2.4 Measurement of L-lactate in plasma

The l-lactate level was assessed in plasma using colorimetric assays according to the manufacturer’s protocol with slight modifications (Lactate Assay Kit, Sigma-Aldrich, Missouri, United States, # MAK064-1KT). A volume of 25 μL of lactate standards or plasma samples was mixed with an equal volume of Master Reaction Mix (comprising 92% lactate assay buffer; 4% lactate probe, and 4% lactate enzyme mix). After a 30-minute incubation, absorbance was measured at 570 nm (A570), using the Cytation 5 Cell Imaging Reader (Agilent BioTek, CA, United States). Lactate levels present in the samples were estimated from the standard curve.

### 2.5 Measurement of L-lactate in blood

L-lactate levels in the blood were measured using blood lactate meter according to the manufacturer’s protocol (Lactate Pro 2, ARKRAY Inc., Kyoto, Japan).

### 2.6 Assessment of neutrophil counts by flow cytometry

Suspended lysed blood cells were centrifuged at 500 × g for 5 min and washed twice with phosphate-buffered saline (PBS). Cells were then suspended in PBS, and neutrophils were detected using a 13-color flow cytometer CytoFLEX system (Beckman Coulter Life Sciences CytoFLEX benchtop flow cytometer) as follows. Suspended cells were incubated in the dark at room temperature for 30 min with CD66b-APC-Alexa Fluor 750 (Beckman Coulter Life Sciences, B08756) to detect neutrophils. After the incubation period, cells were washed with PBS, suspended in 300 μL PBS, and examined by flow cytometry to gate the neutrophil-specific CD66b-APC-Alexa Fluor 750 positive population. A total of 20,000 events were acquired and then analyzed using CytExpert software.

### 2.7 Measurement of extracellular acidification rate (ECAR) in different populations by seahorse XF96 flux analyzer

Extracellular acidification rates (ECAR) in neutrophils, PBMCs, and platelets were assessed using XF analysis (XF96, Seahorse analyzer, Agilent) as described previously (Yasseen et al., 2022). Cells were plated on 96 well format XF plates at a density of 2 × 10^5^ neutrophils/well, 2.5 × 10^5^ PBMCs/well and 20 × 10^6^ Platelets/well in unbuffered DMEM deprived of glucose and pyruvate (DMEM; with 4 mML-glutamine, pH 7.4 at 37°C) for the glycolytic stress tests. Plates were centrifuged at 800 × g for 5 min to form a monolayer of cells in the wells. Plates were then incubated for 30–40 min at 37°C in a non-CO_2_ incubator. A baseline measurement for ECAR was acquired for 30 min. ECAR values were examined following consecutive injections of glucose (5.5 mM), oligomycin (2.5 µM), and 2-deoxy-D-glucose (2-DG) (50 mM). Glycolysis and glycolytic capacity were calculated by subtracting the average rates after the addition of 2-DG from the average rates after the addition of glucose and ATP synthase inhibitor oligomycin. All measurements were normalized to the seeding density before being multiplied by the respective neutrophils count per µL of blood determined by flow cytometry.

### 2.8 Statistical analysis and data presentation

Statistical analysis and data graphing were performed using OriginPro 2022b (OriginLab Corporation, Northampton, United States). Graphical representations of data utilized box-and-whisker plots showing mean ± SD along with the actual scatter of the data points combined with a distribution curve to indicate normality. Density Color Mapping, which assigns color based on the density of points in a two-dimensional scatter plot, was used to infer associative variance and covariance directionality between the two-plotted parameters. Exact *p*-values are given for each comparison on most graphs and in the text. Following tests for normality (Shapiro–Wilk test), variables that passed the normality test were analyzed using the ANOVA test followed by Tukey *post hoc* tests to compare the differences between the three groups, or the independent samples *t*-test, while non-normally distributed data were analyzed using the Mann-Whitney test to compare the differences between two groups. Categorical variables are reported as counts and percentages while continuous variables are expressed as mean ± SD or a median (range). Differences between percentages were assessed by Pearson’s χ^2^ tests or Fisher exact tests when the number of observations per group was less than 5. The χ^2^ tests provided results that tested the hypothesis that mortality and a given variable (e.g., sex or comorbidity) are independent. Linear regression analysis and Pearson correlation coefficients were obtained without asymptotic assumptions. When the *p*-value was less than the significant level of 0.05, significant evidence of an association between mortality and the variable was observed.

## 3 Results

### 3.1 Demographic, clinical, and hematologic characteristics of the studied COVID-19 cohort

The demographic and clinical characteristics of the patients studied are given in Table 1. Participants were divided into two groups: ICU-survivors (ICU-S, n = 36) and ICU-non-survivors (ICU-NS, n = 66). The two groups did not exhibit statistically significant differences between most of the clinical or demographic characteristics except for the blood saturation level (*p* = 0.045). Additionally, the number of patients suffering from diabetes is significantly higher in the ICU-NS group (*p* = 0.008). Meanwhile, the number of patients treated with steroids, remdesivir, hydroxychloroquine, and/or carbapenem antibiotics did not differ significantly between the two groups. Table 2 shows the laboratory results of the participants. When comparing all parameters in the two groups, we observed changes following the same reported trends in our previous studies (Yasseen et al., 2022). Non-significant changes were reported in all the parameters except for a significant decrease in albumin level in the ICU-NS group (*p* = 0.03) and in platelets count (*p* = 0.017). The results also show a significant increase in the white blood cells count (*p* = 0.02), C-reactive protein (*p* = 0.008), and D-Dimer level (*p* = 0.008) in non-survivors.

**TABLE 1:** Demographic and clinical characteristics of the participants.

	ICU-S n (%)	ICU-NS n (%)	*p*-value
Male	23 (63.9%)	34 (51.5%)	0.23
Age	61 (25–77)	66 (17–98)	0.12
sO_2_	94 (59–99)	90 (41–97)	**0.045**
Diabetes	6 (16.7%)	28 (42.4%)	**0.008**
Cardiovascular diseases	11 (30.6%)	26 (39.4%)	0.37
Cancer	4 (11.1%)	3 (4.5%)	0.21
Asthma	2 (5.6%)	3 (4.5%)	0.82
Insulin	6 (16.7%)	16 (24.2%)	0.37
Anticoagulant	16 (44.4%)	34 (51.5%)	0.49
Steroids	11 (30.6%)	31 (47%)	0.11
Hydroxychloroquine	1 (2.87%)	2 (3%)	0.94
IL6 inhibitors	3 (8.3)	5 (7.6%)	0.89
Remdesivir	3 (8.3%)	15 (22.7%)	0.07
Ivermectin	1 (2.8%)	2 (3%)	0.94
Carbapenem antibiotics	11 (30.6%)	32 (48.5%)	0.08
Fluoroquinolone	7 (19.4%)	21 (31.8%)	0.18
Oxazolidinone antibiotic	7 (19.4%)	10 (15.2%)	0.58

Bold values in indicate statistical significance among the groups .

**TABLE 2:** Laboratory results of participants.

	ICU-S	ICU-NS	*p*-value
WBCs (×10^3^/mL)	9.5 (4.4–20.7)	13.2 (4.2–47)	**0.02**
Platelets (10^6^/mL)	274.5 ± 115	211.26 ± 118	**0.017**
Lymphocytes	3.45 (0.48–24.5)	3.4 (0.25–32.5)	0.92
Monocytes	0.99 (0.11–10.2)	1.26 (0.2–12.4)	0.44
INR	1.12 (1–2.86)	1.2 (1–4.4)	0.34
CRP (mg/L)	56.5 (1.9–265)	109.03 (12–305)	**0.008**
D-Dimer (mg/mL)	0.98 (0.17–5.2)	2 (0.19–19.1)	**0.008**
IL-6 (pg/mL)	25 (2.3–1830)	32.4 (1.77–4,500)	0.90
Ferritin	797 (218–2032)	1,148 (139–20000)	0.06
Albumin	2.75 (2.1–3.8)	2.5 (1.6–246)	**0.03**
haemoglobin (g/dL)	11.7 (5.7–15.2)	11.2 (5.5–74)	0.68
ALT (U/L)	19 (8–92)	35 (3–232)	0.23
AST (U/L)	29 (17–40)	37 (12–168)	0.08
Creatinine (mg/dL)	0.96 (0.3–6.85)	1.3 (0.38–6.9)	0.13

Bold values in indicate statistical significance among the groups .

### 3.2 Non-survivors exhibit impaired oxygen delivery and blood acidosis

**FIGURE 1:**
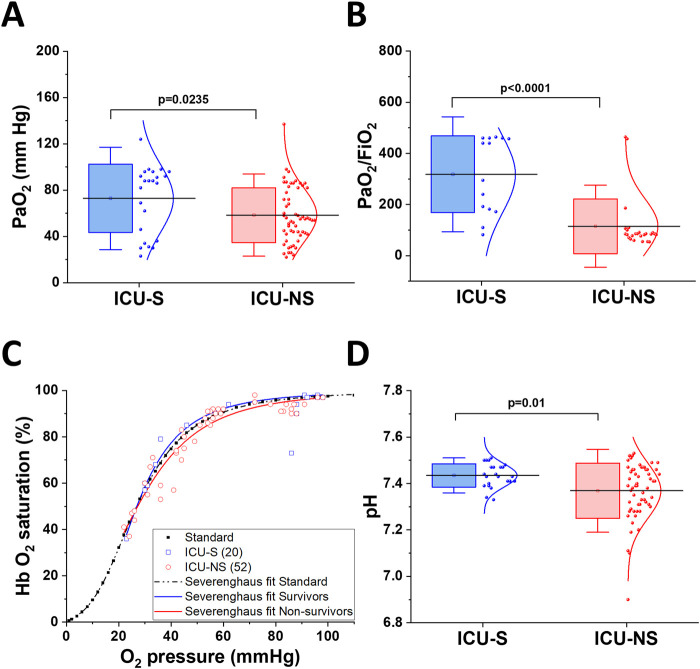
Impaired oxygen delivery in critical COVID-19 patients and blood acidosis. **(A)** A Box plot showing a decrease in arterial oxygen pressure in ICU-NS patients compared to ICU-S (n = 23 and 58 for ICU-S and ICU-NS, respectively). **(B)** Shows a significant decrease in PaO_2_/FiO_2_ ratio in ICU-NS group compared to ICU-S group [(n = 14 and 25 for ICU-S, ICU-NS; respectively)]. **(C)** Graphic depiction of Hb-O_2_ affinity ODC, demonstrating the standard Severinghaus curve (dashed black curve). Values of oxygen saturation plotted *versus* oxygen pressure for ICU-S COVID-19 patients (blue rectangles) and ICU-NS COVID-19 patients (red circles). **(D)** A box plot showing a significant decrease in blood pH in the ICU-NS group when compared to the ICU-S group (n = 23 and 57 for ICU-S and ICU-NS, respectively). All data is plotted as mean ± SD.

A hallmark of COVID-19 infection is hypoxia in critical patients, and our cohort showed decreased partial pressure of oxygen in ICU-NS patients compared to ICU-S (Figure 1A, ICU-S: 72.9 ± 29.5; n = 23, ICU-NS: 58.4 ± 23.7; n = 58. ICU-S vs ICU-NS: *p* = 0.0235). In order to compare our measured parameters with those reported for control subjects, meta-analysis paper was used to extract relevant data (Forrer et al., 2023). When comparing PaO_2_ = 91.5 ± 9.75 for controls with ICU-S and ICU-NS patients, the following statistics were computed: Control vs. ICU-S *p* < 0.0001; Control vs. ICU-NS *p* < 0.0001. Moreover, the PaO_2_/FiO_2_ ratio is frequently used to determine the severity of lung injury in mechanically ventilated patients. Here we show that the PaO_2_/FiO_2_ ratio is remarkably lower in the case of ICU-NS, indicating marked hypoxemia (Figure 1B, ICU-S: 318.1 ± 149.9; n = 14, ICU-NS: 115.1 ± 107.1; n = 25. ICU-S vs ICU-NS: *p* < 0.0001). Oxygen-hemoglobin dissociation curves (ODC) were constructed using ICU-survivors (ICU-S, n = 20) and ICU-non-survivors (ICU-NS, n = 52) COVID-19 patient data and compared to the standard human ODC at T = 37°C, pH = 7.4, created by the Severinghaus model (Figure 1C). First, the theoretical standard human blood ODC was estimated based on the original computations implemented by Severinghaus (1958). A left shift of the Hemoglobin oxygen dissociation curve of the ICU-S group is observed, while the Hemoglobin oxygen dissociation curve of the ICU-NS group is shifted towards higher oxygen pressure to reach the same degree of hemoglobin saturation. These results suggest an exacerbated respiratory dysfunction and relatively impaired gas exchange in ICU-NS COVID-19 patients. Next, we hypothesized that the observed shift in the ODC may result from lowered blood pH during the critical COVID-19 illness, particularly in non-survivors. Analysis of the chemistry laboratory results retrieved during hospitalization revealed that the average blood pH value was indeed lower in ICU-NS (pH = 7.37 ± 0.12, n = 57) relative to ICU-S group (7.43 ± 0.05, n = 23, *p* = 0.01) (Figure 1D). Since the ICU-NS group contained significantly more diabetic comorbodity, the control cohort data extracted yielded mean pH values of 7.41 ± 0.02 with statistical analyses of Control vs. ICU-S *p* < 0.0001; Control vs. ICU-NS *p* < 0.0001. Declines in blood pH can be a result of either respiratory or metabolic acidosis.

### 3.3 Lung damage predicts mortality in critically ill patients with hyperlactatemia

**FIGURE 2:**
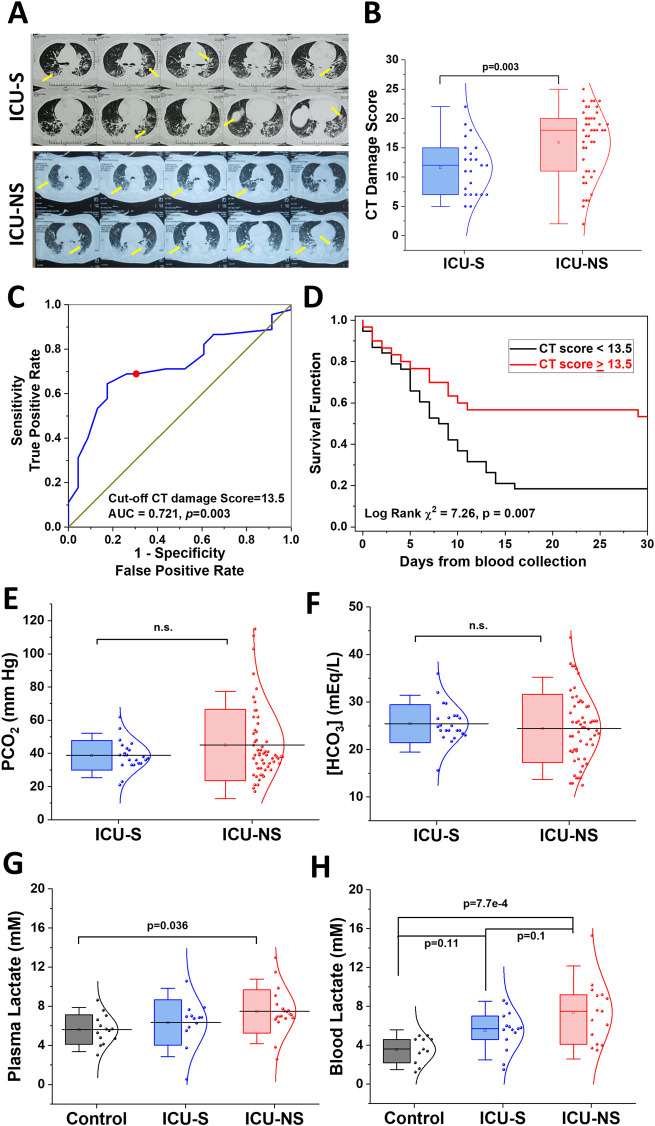
Non-survivors exhibit enhanced lung damage accompanied by metabolic acidosis but not respiratory acidosis. **(A)** Representative chest radiographs for ICU-S and ICU-NS groups with arrows depicting lung damage. **(B)** A box plot showing a significant increase in lung damage in the ICU-NS group relative to the ICU-S group (n = 23 and 45 for ICU-S and ICU-NS, respectively). **(C)** Receiver operating characteristics (ROC) showing optimal sensitivity and specificity of CT lung damage score as predictors of mortality in severe COVID-19 patients, with cut-off values [13.5 (AUC = 0.745, p = 4 × 10^−4^)]. **(D)** Kaplan–Meier estimates of time-to-mortality from blood sample collection during ICU hospitalization. Log-rank Kaplan–Meier survival analyses were carried out to estimate the probability of survival of COVID-19 patients in relation to cutoff thresholds arbitrarily selected as the mean values of the analyzed parameters. Risk Score defined as [CT score], the number of analyzed ICU-COVID-19 patients were 23 ICU-S and 45 ICU-NS. **(E)** Shows box plots of non-significant differences in PCO_2_ (n = 23 and 57), and **(F)** [HCO_3_^−^] (n = 23 and 58) levels between ICU-S and ICU-NS. **(G)** A box plot showing a significant increase in plasma lactate level in the ICU-NS group when compared to the control group (n = 14 and 21 for control, ICU-NS; respectively). **(H)** A box plot showing a significant increase in blood lactate level in the ICU-NS group when compared to the control group (n = 11 and 15 for control, ICU-NS; respectively). All data is plotted as mean ± SD. N.s, not significant.

SARS-CoV-2 infection usually leads to complications such as pneumonia, and in severe cases, ARDS and sepsis, which are now known to leave lasting damage to the lungs including interstitial lung disease (Stewart et al., 2022). We analyzed the extent of lung damage in a subset of the studied cohort by analyzing the retrieved chest radiographs for 23 ICU-S and 45 ICU-NS as described in Methods; representative images are given in Figure 2A. ICU-NS group showed significantly greater lung damage with a mean score ±SD = 15.93 ± 5.87 vs 11.61 ± 4.68 for ICU-S group (*p* = 0.003), Figure 2B. We then proceeded to explore the prognostic power of the CT score as a mortality predictor. We started by generating a receiver-operating characteristic (ROC) curve for the prediction of mortality in terms of the CT score (Figure 2C). This analysis established a CT damage score cut-off value of 13.5 (AUC = 0.721, *p* = 0.003) as a stratifying parameter for mortality prediction in the subsequent Kaplan-Meier survival analysis (Figure 2D). CT lung damage score ≥13.5 was subsequently used as a predictor of mortality within 20 days of image acquisition (31 out of 38, 81.5%; Log Rank χ^2^ = 7.26, *p* = 0.007). Respiratory acidosis results from hypoventilation and subsequent elevation of PCO_2_. Laboratory results obtained for participants at the site of sample collections during hospitalization revealed non-significant differences in PCO_2_ (Figure 2E) (ICU-S, n = 23; ICU-NS, n = 57), or in [HCO_3_^−^] levels (Figure 2F) (ICU-S, n = 23; ICU-NS, n = 58) between ICU-survivors and ICU-non-survivors. This confirms that the decline in pH is not due to respiratory acidosis or metabolic acidosis from enhanced loss of HCO_3_^−^. To answer the remaining arm of metabolic acidosis, we investigated lactate levels in the plasma and blood of ICU patients. Plasma levels of lactate, determined colorimetrically, were significantly higher in ICU-NS patients than in controls (Control: 5.62 ± 1.5; n = 14, ICU-S: 6.34 ± 2.32; n = 13, ICU-NS: 7.47 ± 2.2; n = 21. ICU-S vs Control: *p* = 0.64; ICU-NS vs Control: *p* = 0.036; ICU-S vs ICU-NS: *p* = 0.27), indicating a marked lactic acidosis in the non-survivors group (Figure 2G). To further confirm these findings, we also measured blood lactate levels in stored blood samples from a subset of controls, ICU-S and ICU-NS subjects using a blood lactate meter (Control: 3.55 ± 1.4; n = 11, ICU-S: 5.51 ± 2.01; n = 15, ICU-NS: 7.37 ± 3.19; n = 15. ICU-S vs Control: *p* = 0.11; ICU-NS vs Control: *p* = 7.7 × 10^−4^; ICU-S vs ICU-NS: *p* = 0.1). Our results showed that blood lactate levels were significantly elevated in the ICU-NS group when compared with controls (Figure 2H). Moreover, we reanalyzed our data reporting lactate levels in both blood and plasma while taking into account the discrepancy between the number of diabetic patients in each group. When we compared their levels of lactate in either plasma or whole blood, we found no statistical difference between those groups. Specifically, for plasma lactate levels: ICU-S = 6.2 ± 2.5 (n = 11); ICU-S DM = 7.3 ± 0.79 (n = 2); *p*-value = 0.88; ICU-NS = 8.4 ± 1.94 (n = 13); ICU-NS DM = 5.9 ± 1.75 (n = 8); *p*-value = 0.057. As for blood lactate levels: ICU-S = 5.49 ± 2.17 (n = 13); ICU-S DM = 5.65 ± 0.07 (n = 2); *p*-value = 0.99; ICU-NS = 6.75 ± 2.78 (n = 8); ICU-NS DM = 8.07 ± 3.70 (n = 7); *p*-value = 0.78. In conclusion, diabetes mellitus was not a significant confounding factor regarding blood and plasma lactate content.

### 3.4 Elevated counts of the glycolytic neutrophils contribute to lactate production in nonsurvivors

**FIGURE 3:**
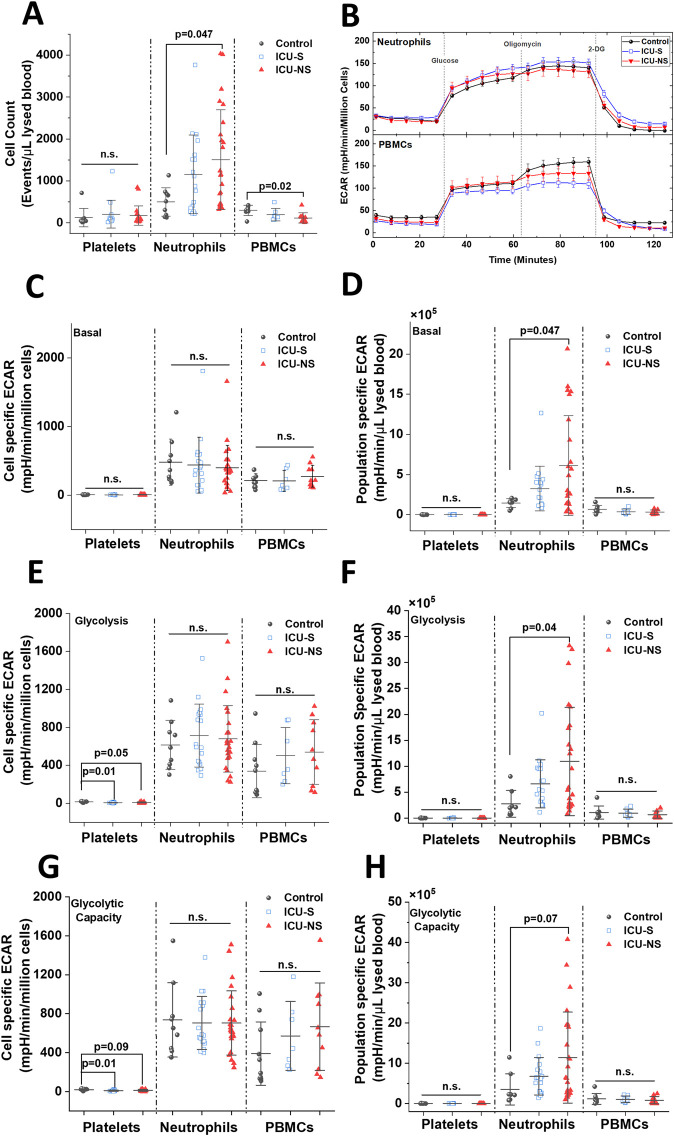
Non-survivors display significant enhancement in global neutrophil glycolysis, but not in neutrophil glycolysis normalized to neutrophil counts in the blood. **(A)** A box plot comparing neutrophils (n = 9, 18, and 24 for control, ICU-S, and ICU-NS, respectively), platelets (n = 9, 13, and 20 for control, ICU-S, and ICU-NS, respectively), PBMCs (n = 8, 7 and 10 for control, ICU-S and ICU-NS, respectively) counts/µL of lysed blood in all of the studied subjects. **(B)** Representative seahorse traces for ECAR (glycolytic flux) were measured in neutrophils freshly isolated from control, ICU-S, and ICU-NS and normalized to the number of seeded neutrophils. When basal ECAR, glycolytic metabolism, and glycolytic capacity normalized to seeded cell counts for platelets (n = 9, 13 and 23 for control, ICU-S and ICU-NS, respectively), neutrophils (n = 9, 18, and 24 for control, ICU-S and ICU-NS, respectively) and PBMCs (n = 10, 7 and 10 for control, ICU-S and ICU-NS, respectively) were compared for all groups, no statistical significant differences were detected **(C, E, G)**. When population-specific basal ECAR per µL lysed blood **(D)**, glycolytic metabolism **(F)**, and glycolytic capacity **(H)** for platelets (n = 9, 13 and 23 for control, ICU-S and ICU-NS, respectively), neutrophils (n = 8, 18 and 24 for control, ICU-S and ICU-NS, respectively) and PBMCs (n = 8, 7 and 10 for control, ICU-S and ICU-NS, respectively) were compared for all groups, ICU-NS neutrophils displayed significant increase relative to ICU-S and control groups in basal and glycolytic metabolism and a trend in glycolytic capacity. All data is plotted as mean ± SD. N.s., not significant.

We asked if and how leukocytes contribute to the observed increase in blood/plasma lactate levels and lowered pH, especially in non-survivors (Figure 3). To identify potential contributors to lactic acidosis in circulating blood, we assessed and compared glycolytic activities in isolated neutrophils, platelets, and peripheral blood mononuclear cells (PBMCs) from the current cohort of critically ill patients relative to the control group. First, group-dependent changes in cell numbers in these three cell types were assessed using flow cytometry to show that only neutrophil counts exhibit a remarkable increase with severe COVID-19 infection (Figure 3A, Control: 499.32 ± 338.9; n = 9, ICU-S: 1,156.42 ± 938.9; n = 18, ICU-NS: 1,507.42 ± 1,191.19; n = 24. ICU-S vs Control: *p* = 0.26; ICU-NS vs Control: *p* = 0.04; ICU-S vs ICU-NS: *p* = 0.50). In fact, PBMCs showed a significant decrease in non-survivors, Figure 3A (Control: 300.13 ± 122.46; n = 8, ICU-S: 195.71 ± 145.47; n = 7, ICU-NS: 117.34 ± 122.75; n = 10. ICU-S vs Control: *p* = 0.28 ICU-NS vs Control: *p* = 0.02; ICU-S vs ICU-NS: *p* = 0.45).

Traces of Seahorse metabolic analysis of freshly isolated neutrophils from subsets representing all groups demonstrate that the extracellular acidification rate by individual neutrophils is independent of mortality outcome (Figure 3B). Generally, for both neutrophils and PBMCs, basal ECAR, glycolytic metabolism, and glycolytic capacity normalized to seeded cell counts did not show any significant differences among all groups (Figure 3, panels C, E, G). Only platelets exhibited significantly reduced glycolytic flux in critically ill COVID-19 patients, which confirms our recently published data (Yasseen et al., 2022). However, a significant increase in population-specific basal ECAR per µL lysed blood (Figure 3D, Control: 1.44 × 10^5^ ± 0.54 × 10^5^; n = 8, ICU-S: 3.26 × 10^5^ ± 2.78 × 10^5^; n = 18, ICU-NS: 6.1 × 10^5^ ± 6.2 × 10^5^; n = 24. ICU-S vs Control: *p* = 0.63; ICU-NS vs Control: *p* = 0.047; ICU-S vs ICU-NS: *p* = 0.13), glucose-supplemented glycolytic metabolism (Figure 3F, Control: 2.76 × 10^5^ ± 2.57 × 10^5^; n = 8, ICU-S: 6.62 × 10^5^ ± 4.62 × 10^5^; n = 18, ICU-NS: 10.94 × 10^5^ ± 10.44 × 10^5^; n = 24. ICU-S vs Control: *p* = 0.49; ICU-NS vs Control: *p* = 0.04; ICU-S vs ICU-NS: *p* = 0.19), and a trend in glycolytic capacity when mitochondria are inhibited with oligomycin (Figure 3H, Control: 3.53 × 10^5^ ± 3.86 × 10^5^; n = 8, ICU-S: 6.79 × 10^5^ ± 4.65 × 10^5^; n = 18, ICU-NS: 11.43 × 10^5^ ± 11.3 × 10^5^; n = 24. ICU-S vs Control: *p* = 0.64; ICU-NS vs Control: *p* = 0.07; ICU-S vs ICU-NS: *p* = 0.2) have been detected in ICU-NS neutrophils relative to ICU-S and control groups. These results suggest that the observed increase in glycolysis in ICU-NS neutrophils is driven by a rise in neutrophil counts rather than a metabolic shift in individual neutrophils.

### 3.5 Neutrophil counts associated with blood pH and plasma lactate levels

**FIGURE 4:**
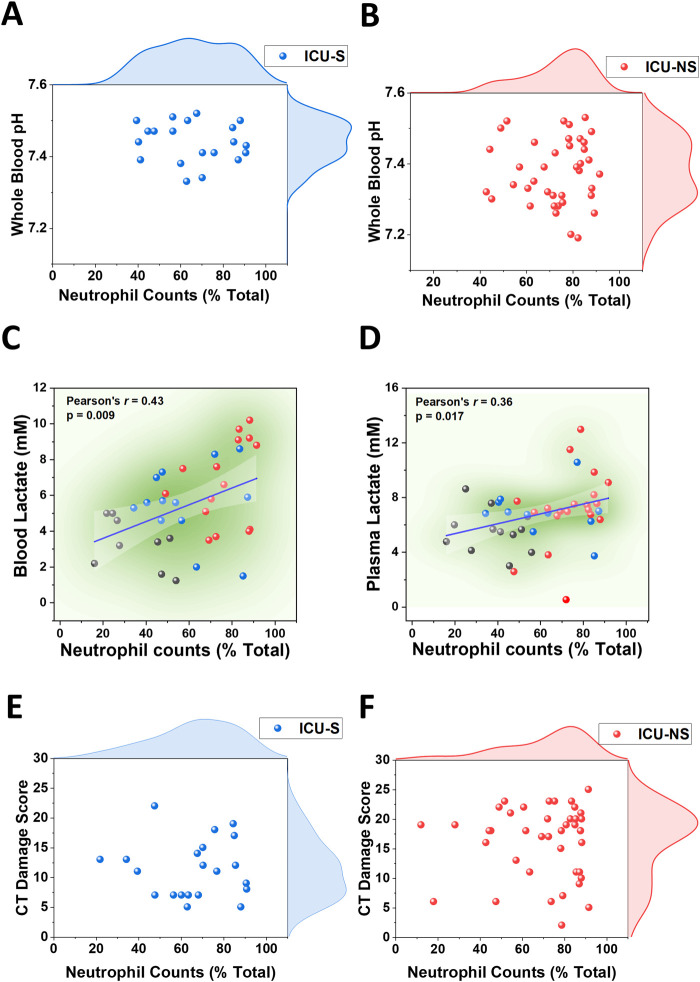
Associations of neutrophilia with blood acidosis, and hyperlactatemia in blood and plasma in ICU-COVID-19 subjects. **(A)** Scatter plot with the distribution of circulating neutrophil counts and whole blood pH in ICU-S patients (n = 20). **(B)** Scatter plot with the distribution of circulating neutrophil counts and whole blood pH in ICU-NS patients (n = 40). Linear correlation between circulating neutrophil counts and blood lactate levels **(C)** and plasma lactate levels **(D)** in (controls (black circles), ICU-S (blue circles), and ICU-NS (red circles) groups (n = 9, 13 and 15 for blood and n = 12, 11 and 20 for plasma, respectively). **(E)** Scatter plot with the distribution of circulating neutrophil counts and CT damage score in ICU-S patients (n = 21). **(F)** Scatter plot with the distribution of circulating neutrophil counts and CT damage score in ICU-NS patients (n = 41).

We then investigated the relationship between neutrophil counts and lactic acidosis in COVID-19 ICU-patients. A rise in lactate levels that coincides with increased neutrophil counts in the circulation and inflamed tissues has been demonstrated in multiple pathological conditions including shock, sepsis, and ischemia (Andersen et al., 2013) but not in COVID-19. Here, we compared neutrophil counts with blood pH in critically ill patients as shown in the scatter and distribution plots (Figure 4A for ICU-S; Figure 4B for ICU-NS), which shows an increased tendency toward lower pH in patients exhibiting greater neutrophil counts, especially in ICU-NS group. In Figure 4C we show that blood lactate levels positively correlate with neutrophil counts (Pearson’s r = 0.43, *p* = 0.009), a trend that was also conserved for plasma lactate levels albeit with weaker but significant correlation (Figure 4D, Pearson’s r = 0.36, *p* = 0.017). These results further suggest that neutrophilia are significantly contributing to lactic acidosis in critically ill patients. These results recalled an important question: Is there a link between neutrophilia and lung damage? To answer this question, we plotted the CT damage score as a function of neutrophil counts (Figure 4E for ICU-S; Figure 4F for ICU-NS). Although no statistically significant correlation has been observed to linearly connect these parameters, a qualitative association can be seen that indicates distribution at higher values of both neutrophil counts and CT damage scores in the ICU-NS group. This suggests a subtle association between neutrophilia and COVID-19-caused lung damage.

## 4 Discussion

Our COVID-19-related research efforts have focused on a specific question: Why do differential mortality outcomes arise in a homogeneous cohort of intensive care patients with severe COVID-19 who exhibit similar demographic and clinical characteristics? We have implicated neutrophils in extensive oxidative damage of circulating human serum albumin (Badawy et al., 2021) and in platelets activation and hypercoagulability (Yasseen et al., 2022), particularly in patients with poor mortality outcomes. Neutrophilia in critical COVID-19 illness has been identified as one of the earliest risk factors associated with mortality (Qin et al., 2020). In this study, we investigate the contribution of acidosis in impairing oxygen delivery in COVID-19 critical patients by exploring its sources, focusing on lung damage and lactate production through glycolysis in blood cells.

Numerous studies have provided evidence that COVID-19 severity and high in-hospital mortality are often associated with severe hypoxemia (Xie et al., 2020). In this study, we confirm a decline in partial pressure of oxygen (PaO_2_) and in the PaO_2_/FiO_2_ ratio, which are indicative of lung injury severity in ICU-NS patients compared to ICU-S patients. The hypoxemia may be due to a decrease in oxygen delivery and oxygen affinity by RBCs or hemoglobin specifically. We also show that the oxygen dissociation curve of the ICU-NS group exhibits a rightward shift compared to that under standard conditions or in the ICU-S groups. Published data on ODC curves stratified by mortality outcomes in hospitalized critically ill COVID-19 patients are scarce [reviewed recently in (Böning et al., 2023)], but the few identified studies suggest that left-shifted ODCs are usually associated with better prognoses (Valle et al., 2022). Recently, it has been proposed that the ODC in critically ill COVID-19 patients displays a left shift compared to the standard ODC (Ceruti et al., 2022) or to patients suffering from hypoxia due to other respiratory disorders (Vogel et al., 2020). Indeed, our results indicate that the ODC of ICU-survivors COVID-19 patients are shifted to the left compared with standard conditions, while the ODC of ICU-non-survivors is shifted toward higher oxygen pressures to achieve the same degree of hemoglobin saturation. These align with the study by Ceruti et al., which demonstrated an absence of a left shift in critically ill COVID-19 patients with poor outcomes compared to those discharged from the ICU (Ceruti et al., 2022). Among the possible reasons for the rightward shift of the hemoglobin oxygen dissociation curve are increases in PCO_2_ and decreases in pH, a phenomenon known as the Verigo-Bohr effect.

Our analysis indicates that the blood of non-survivors is generally more acidic, with lower pH values in ICU-NS patients compared to ICU-S patients. Our results are consistent with the study by Kieninger et al., which identified low blood pH as a significant prognostic factor for in-hospital mortality in critically ill COVID-19 patients (Kieninger et al., 2021). The observed drop in blood pH can result from either respiratory or metabolic acidosis. Respiratory acidosis is caused by hypoventilation, and subsequent elevation of PCO_2_, whereas metabolic acidosis is the result of excessive generation of acid production, most commonly lactic acid and/or enhanced loss of HCO_3_^−^ (Kraut and Madias, 2010). In this context, we examined whether mortality is associated with increased blood PCO_2_ or HCO_3_^−^ levels. Our results showed no significant differences in PCO_2_ or HCO_3_^−^ levels between ICU-survivors and ICU-non-survivors, despite significant lung damage indicated by CT scores in ICU-NS compared to ICU-S patients. Ruling out respiratory acidosis and changes in bicarbonate metabolic contribution, we investigated lactate levels in our cohort. In contrast, ICU-non-survivors displayed significantly higher levels of plasma and blood lactate compared with ICU-survivors, pointing to a significant lactic acidosis in critically ill COVID-19 patients with fatal outcomes. Our findings are consistent with other studies showing that non-survivor COVID-19 patients exhibited greater levels of blood lactate compared with survivors [reviewed in: (Carpenè et al., 2022)]. Moreover, numerous studies reported hyperlactatemia in ICU-non-survivors patients with significant mortality prediction scores between survivors and non-survivors (Carpenè et al., 2022). According to the Verigo-Bohr effect, a slight decline in blood pH causes a significant reduction in oxygen saturation. With the progression of oxygen insufficiency, acidosis is exacerbated and thus continues to hinder oxygen delivery to peripheral tissues (Nechipurenko et al., 2021). Elevated lactate feeds back into this cycle by lowering the average pH and reducing the oxygen-carrying capacity of hemoglobin (Haller et al., 2021).

However, the sources underlying the overproduction of lactate leading to lactic acidosis in critically ill COVID-19 patients in relation to mortality outcomes are still unexplored experimentally. We attempted to shed some light on the source(s) of circulating lactate in the studied patients. Under physiological conditions, red (RBCs) and white (WBCs) blood cells are minor sources of lactate. Although RBCs exclusively rely on glycolysis for their bioenergetic demands, we ruled out RBCs as an important player in the observed metabolic acidosis because RBCs were shown to act as a lactate sink in COVID-19 (Mullen et al., 2022). Furthermore, we have previously shown that glycolytic activities in platelets actually decrease with severity (Yasseen et al., 2022). However, the situation with the WBCs might be different during immune activation and inflammation, presumably due to over-activation and metabolic rewiring of the activated WBCs. Dramatic increases in neutrophil counts in ICU-hospitalized patients enticed us to explore the subsequent implications of their metabolic remodeling while hypothesizing a role for lactate overproduction by glycolytic neutrophils in the well-documented impairment of oxygen delivery and hypoxemia. We thus characterized glycolytic activities in platelets, neutrophils, and PBMCs freshly isolated in representative groups of controls, survivors, and non-survivors of COVID-19 patients. Surprisingly, we found that COVID-19 severity and mortality are not associated with glycolytic shifts in any of the studied cell types except for a pronounced reduction in glycolytic fluxes in platelets. However, when we considered the changes in cell counts for each blood cell type, we concluded that neutrophilia significantly contributes to lactate production in non-survivors. We also demonstrated that elevated plasma and blood lactate levels, as well as blood acidity, are significantly correlated with increased neutrophil counts in non-survivors COVID-19 patients.

We believe blood and plasma lactate levels play a significant part in the complex etiology of the mortality outcome in the most severe COVID-19 infections. However, it is not clear if remarkably increased lactate levels are mortality correlates or actually contribute to the pathological factors leading to death outcomes in those patients. When the production of lactic acid increases while its clearance decreases, the clinical severity intensifies. Elevated lactic acid levels can significantly impact hemodynamics and may result in fatal outcomes (Kimmoun et al., 2015). Serum lactate serves as both a risk marker and a therapeutic target (Green et al., 2011). Higher serum lactate levels and prolonged normalization times are associated with an increased risk of mortality independent of organ failure (Mikkelsen et al., 2009). Furthermore, serum lactate was found to be a consistent mortality risk factor independent of the presence of acid-base disorders, inflammation, malnutrition, and renal or hepatic dysfunction in the pediatric ICU-patients (Yue et al., 2022). Our combined results indicate that lactate levels in the ICU-NS group are significantly greater than in the control group’s blood and plasma but exhibit a somewhat less magnified increase when compared with the ICU-S group. We believe that such an increase would be more statistically significant with bigger cohorts. Collectively, the current findings suggest that neutrophilia is contributing to lactic acidosis, impaired oxygen delivery, and lung damage in critically ill patients. These findings, despite being in need for further confirmation, may represent an additional step toward understanding the pathophysiology of lactic acidosis detected in critically ill COVID-19 patients with fatal outcomes.

## 5 Data limitations and perspectives

One of the limitations of this study is that lactate release by damaged organs was not considered as a substantial source of lactate and it may indeed be contributing to the lactate production. However, the current work is not remote from a recent report in the context of sepsis where hyperlactatemia is a mortality risk factor irrespective of organ failure (Mikkelsen et al., 2009). Indeed, correlations between neutrophil counts with blood pH, blood and plasma lactate levels, and even CT damage confirm such an assessment.

## Data Availability

The original contributions presented in the study are included in the article/supplementary material, further inquiries can be directed to the corresponding author.

## Abbreviations

ICU-S, Intensive Care Unit-Survivors; ICU-NS, Intensive Care Unit-Non-Survivors; CT, Computed Tomography; PBS, Phosphate Buffer Saline; PBMCs, Peripheral Blood Mononuclear cells; ECAR, Extracellular acidification rates; ODC, Oxygen Dissociation Curve; RBCs, Red Blood Cells; WBCs, White Blood Cells.
